# Serum hepatitis B virus RNA detectability, composition and clinical significance in patients with ab initio hepatitis B e antigen negative chronic hepatitis B

**DOI:** 10.1186/s12985-022-01749-7

**Published:** 2022-01-29

**Authors:** Andreas Laras, Margarita Papatheodoridi, Eleni Panopoulou, George V. Papatheodoridis, Stephanos J. Hadziyannis, Emilia Hadziyannis

**Affiliations:** 1 2nd Department of Medicine and Laboratory, Hippokration General Hospital, National and Kapodistrian University of Athens, School of Medicine, Vas. Sophias 114, 11527 Athens, Greece; 2 Department of Gastroenterology, General Hospital of Athens “Laiko”, National and Kapodistrian University of Athens, School of Medicine, Athens, Greece

**Keywords:** Hepatitis B virus, HBV RNA, Pregenomic RNA, HBeAg negative, Treatment discontinuation, Biomarker

## Abstract

**Background:**

Serum hepatitis B virus (HBV) RNA is a surrogate biomarker for intrahepatic covalently closed circular DNA (cccDNA) transcriptional activity and persistence. In this retrospective study, we investigated its presence, levels and composition in ab initio Hepatitis B e antigen (HBeAg) negative chronically infected patients and examined possible associations with disease activity and the outcome of nucleos(t)ide analogue (NA) discontinuation.

**Methods:**

We developed a sensitive real time polymerase chain reaction (RT-PCR) for the specific detection of HBV pregenomic RNA (pgRNA) and precore (preC) mRNA and analyzed 220 serum specimens, 160 under NA treatment, from 116 Greek patients initially negative for HBeAg.

**Results:**

HBV pgRNA was detected in 31% and preC mRNA in 15% of samples, at lower levels representing a small fraction (3.4%) of total core promoter produced transcripts. In the absence of NAs, pgRNA was detected in 57% of samples with median value of 5.19 (2.61–8.35) log_10_ cp/mL, at lower levels than HBV DNA and correlated significantly with ALT (r = 0.764) and serum HBV DNA (r = 0.906). A wide range of HBV DNA/pgRNA ratio was observed with significant inter- and intra-patient variation. During NA treatment, pgRNA displayed low detectability (22%) and variable levels, median 3.97 (2.30– 8.13) log_10_ cp/mL, as well as, a significant inverse correlation with the duration of treatment (r = − 0.346, *p* < 0.01). In 74 events of NA discontinuation, end-of-treatment pgRNA-positive compared to pgRNA-negative cases, experienced more frequently virological (*p* = 0.016) and clinical (*p* = 0.011) relapse.

**Conclusions:**

In genotype D ab initio HBeAg negative patients, serum HBV RNA is primarily composed of pgRNA plus a minor fraction of preC mRNA transcripts. Serum pgRNA is associated with disease activity, suggesting lysis of infected hepatocytes as a possible source of serum HBV RNA in untreated patients and in the early phase of NA treatment. During long term NA treatment, detectable serum pgRNA predicts viral rebound and clinical relapse following treatment discontinuation and may thus serve as a marker for the decision of cessation of therapy.

## Introduction

Hepatitis B virus (HBV) is a major cause of serious chronic liver disease including chronic hepatitis B (CHB), cirrhosis and hepatocellular carcinoma (HCC) [1]. In the Mediterranean region, HBV infection is mainly attributed to HBV genotype D which displays distinct viral characteristics and clinical course [2, 3]. Specifically in Greece, the predominant HBV genotype D harbours mutations in the precore and basic core promoter (CP) regions, hepatitis B e antigen (HBeAg) clearance is commonly silent, HBeAg seroconvertion occurs early in life before adulthood and chronic HBV infection is usually detected in the HBeAg negative phase, either as active hepatitis or in the inactive carrier (IC) state [4]. The vast majority of patients with HBeAg negative CHB are treated with nucleos(t)ide analogues (NAs), which inhibit viral replication without directly targeting the key molecule in HBV life cycle, the covalently closed circular DNA (cccDNA) residing in the nucleus of infected hepatocytes [5].

HBV cccDNA directs the generation of viral nucleic acids and gene products required for replication and virion production and is responsible for viral persistence in the natural course and during antiviral treatment [5, 6]. The cccDNA nuclear episome serves as the template for transcription by host enzymes, producing mRNA transcripts translated into viral proteins and the viral RNA pregenome. The 3.5-kb pregenomic RNA (pgRNA) is produced by the HBV core promoter which also guides the synthesis of the slightly (15–35 bp) longer precore (preC) mRNA. Despite their homology, pgRNA is selectively encapsidated and reverse transcribed inside nucleocapsids. Partial synthesis of variable length positive DNA strand leads to the production of mature viral particles, containing the relaxed circular (rc) DNA genome, secreted as mature virions to the blood stream or recycled to the nucleus to increase the available cccDNA pool [6].

Since long-term NA treatment does not directly affect the transcriptional activity of cccDNA [7], production of pgRNA, viral mRNAs and proteins continues from residual intrahepatic cccDNA, accumulated pgRNA is encapsidated and may illegitimately mature and be exported in virion-like particles [8, 9]. Direct monitoring of intrahepatic cccDNA has been proven clinically impractical due to the invasive process of liver biopsy and technically challenging, especially in HBeAg negative patients, where long-term NA treatment results in its drastic reduction at levels bordering the sensitivity of available assays [10, 11]. In recent years, an increasing number of studies have shown that HBV RNA is detectable in the serum of both treated and untreated patients and its clinical use as a surrogate marker for cccDNA activity has been proposed [12, 13]. However, data concerning genotype D ab initio HBeAg negative patients, either under antiviral treatment or during the natural course of infection remain limited.

We developed a sensitive real time polymerase chain reaction (RT-PCR) assay for the specific detection of serum HBV pgRNA and preC mRNA and investigated serum HBV RNA detectability, composition and levels in HBeAg negative subjects as well as their association with disease activity and prediction of the outcome of NA discontinuation.

## Materials and methods

### Patients and samples

In this retrospective study we examined 220 serum samples, collected between 2003 and 2018, from 116 Greek patients with HBeAg negative CHB followed at two Hepatology Outpatient Clinics. Informed consent was obtained from all subjects. The study was conducted according to the guidelines of the Declaration of Helsinki and approved by the local institution (protocol code 2601, date of approval 23-3-2016). Patients with cirrhosis or HCC as well as with Hepatitis D Virus, Hepatitis C Virus and Human Immunodeficiency Virus coinfection were excluded. The main patient characteristics are presented in Table 1. Additionally, 24 samples, serially obtained from two genotype A HBeAg positive NA treated CHB patients, were examined for comparison. All samples were stored in aliquots at − 80 °C and thawed once for analysis.

**Table 1:** Clinical characteristics of HBeAg negative patients included in this study

All HBeAg negative patients, n (samples, n)	116 (220)
Age at sampling time, years	63 (25–86)
Sex, Male / Female	82 / 34
HBV DNA positive, n (samples, n)	37 (60)
Serum HBV DNA levels, log_10_ copies/mL	5.9 (2–9.7)
HBsAg levels, log_10_IU/mL	2.6 (0–4.6)
ALT levels (IU/L)	24 (10–2130)
Patients not under treatment, n (samples, n)	37* (60)
Patients on nucleos(t)ide analogues (NA), n (samples, n)	103* (160)
Duration of NA treatment at sampling, months	74 (1–220)
Patients who stopped NAs, n (EOT samples, n)	73* (74) **
Duration of NA treatment, months	75 (36–220)
Follow-up after NA discontinuation, months	15 (1–96)
Virological relapse within 1st year, n	42
Clinical relapse within 1st year, n	21
Retreatment within 1st year, n	14
Functional cure in long term follow-up	33

All quantitative variables are expressed as median (min–max) values

*Same patients are included in different subcategories thus numbers do not add up

**One patient discontinued NAs twice

### Clinical laboratory measurements

At collection, biochemical tests were performed by standard laboratory procedures. Hepatitis B surface antigen (HBsAg) levels were quantified (qHBsAg) using the Elecsys HBsAg quant Assay (Roche Diagnostics GmbH, Mannheim, Germany). In samples with qHBsAg titer below the threshold of the assay (0.05 IU/mL), HBsAg clearance was verified with the qualitative Elecsys HBsAg test with analytical sensitivity for HBV subtype AY ≤ 0.04 U/mL. The same platform was used for the detection of serological markers including HBeAg and anti-HBe. HBV genotype was determined by direct sequencing as described previously [14]. HBV DNA was measured by RT-PCR using the Roche LighCycler, lower quantification limit (LQD) at 125 (2.1 log_10_) cp/mL of serum, both at the time of visit and HBV RNA analysis [15].

### Serum HBV RNA quantification

HBV RNA was analyzed by a transcript specific RT-PCR which allows the specific detection and quantification of preC mRNA and simultaneously monitors total CP-directed transcription (preC mRNA plus pgRNA). We modified our previous methodology for intrahepatic HBV pgRNA in order to obtain the desired specificity and sensitivity in serum [15].

RNA was extracted from 560 μL serum using the QIAamp Viral RNA Mini Kit (Qiagen, Germany). An enzymatic treatment by on-column treatment with RNase-Free DNase (Qiagen) was included. Antisense primer BC1 (5’-GGAAAGAAGTCAGAAGGCAA, nt1974-1955) and QuantiTect Reverse Transcription Kit (Qiagen), which incorporates gDNA Wipeout for further removal of contaminating DNA, were used for cDNA synthesis. Τhe cDNA product was used in two separate amplification reactions with BC1 as the common 3′ primer and 5′ primers, PCP (5′-GGTCTGCGCACCAGCACC nt1796-1813) for the detection of preC mRNA and PGP (5′-CACCTCTGCCTAATCATC nt1826-1843) for total CP-directed transcription [15]. Primers were positioned in highly conserved regions. The levels of pgRNA were calculated by subtracting preC mRNA from total CP-directed transcription measured in each sample. DNA contamination was monitored by testing extracted RNA for total CP-directed transcription and/or the cDNA product with M3 (5′-CTGGGAGGAGTTGGGGGAGGAGATT nt1730-1754) as a 5′ primer, located upstream from either preC mRNA or pgRNA start sites.

The specificity and sensitivity of the methodology have been described previously [14, 15]. The inclusion of two distinct methods for DNA removal, effectively eliminated all traces of contamination. The lower limit of detection (LLD) for CP-directed transcription was determined at 250 (2.40 log_10_) copies per mL (cp/mL) and the LQD at 350 (2.54 log_10_) cp/mL of serum. The LQD for preC mRNA was 1050 (3.02 log_10_) cp/mL.

### Statistical analysis

Quantitative variables are presented by median values and range. For measurable but not quantifiable pgRNA, the value of 200 cp/mL was used. Statistical comparisons between two groups of patients were performed by Wilcoxon-Mann–Whitney test. Categorical variables were summarized as percentages. The relationship between categorical values was examined by Fisher’s exact test. Spearman’s correlation was used to evaluate probable correlations between parameters as HBV nucleic acids, alanine aminotransferase (ALT), age and treatment duration. Cox proportional hazards regression model was used to estimate the effect HBV RNA and other variables on the probability of virological (VR) and clinical relapse (CR) or HBsAg clearance after NA discontinuation. For logarithmic values, the value 0 was used for negative logarithms and non-detectable levels. Data were analysed using the SPSS 25.0 software (SPSS, Chicago, Illinois, USA). A *p* value of < 0.05 was considered to be statistically significant.

## Results

HBV pgRNA was detectable in 69 (31%) of 220 samples, in 31/116 (26.7%) patients, median 4.70 (range 2.30–8.35) log_10_ cp/mL when measurable. In addition, preC mRNA was detected in 33 (15%) samples, 14/116 (12%) patients, median 3.89 (3.03–7.16) log_10_ cp/ml when detected, representing a small fraction of total CP-directed transcription, median 3.4% (0.01–18.1).

### Patients not receiving NA treatment

**Fig. 1:**
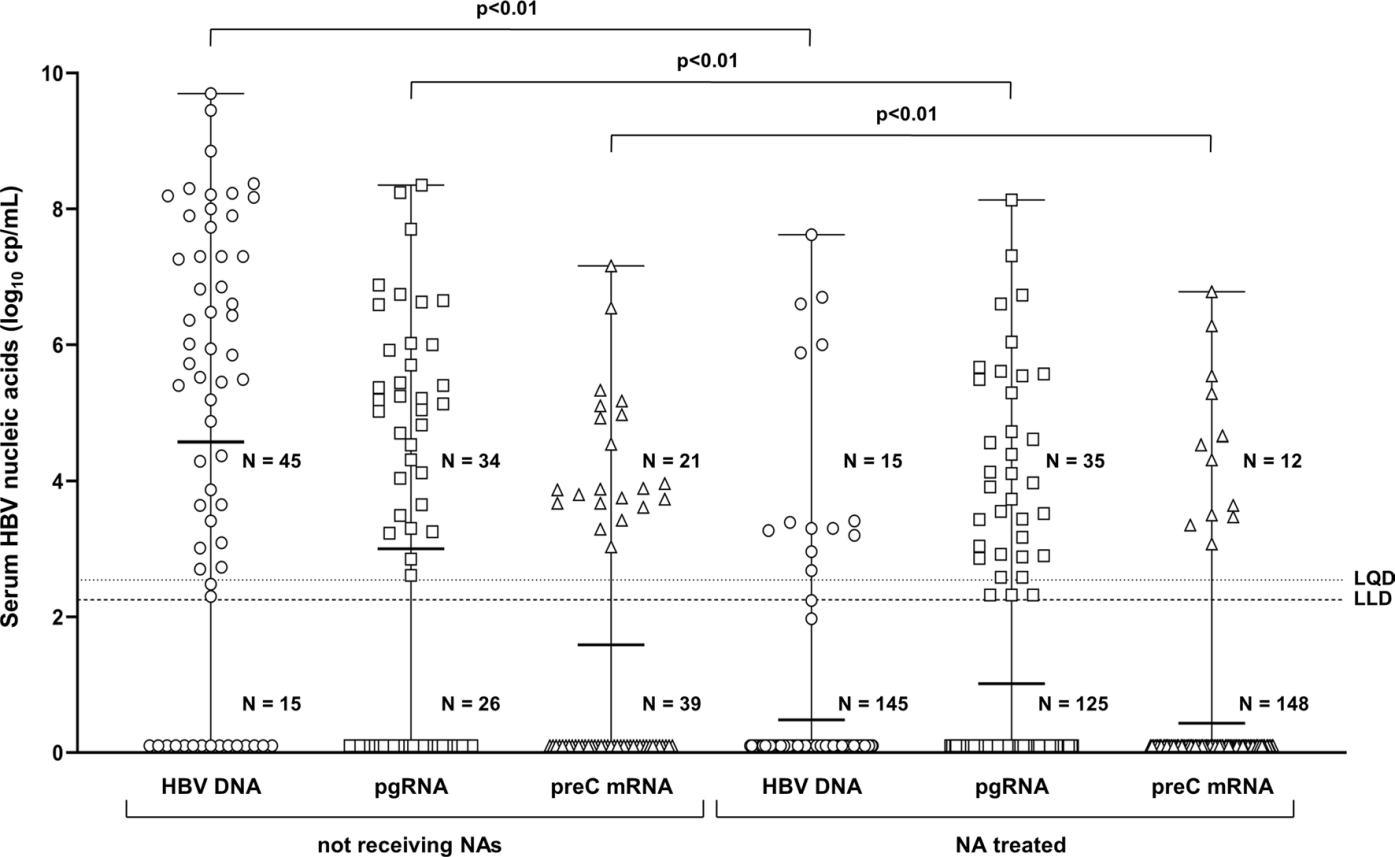
Serum HBV nucleic acid levels in HBeAg negative patients. Serum HBV DNA (circles), pgRNA (squares) and preC mRNA (triangles) levels in patients not receiving any treatment (N = 60) and patients treated with nucleos(t)ide analogues (N = 160). Serum HBV RNA LQD and LLD indicated with dotted lines

Serum HBV RNA was detected in 34 of 60 (56.7%) samples, with pgRNA median levels 5.19 (2.61–8.35) log_10_ cp/mL. PreC mRNA was present in 21/60 (35%) samples with median levels 3.87 (3.03–7.16) log_10_ cp/mL (Fig. 1).

**Fig. 2:**
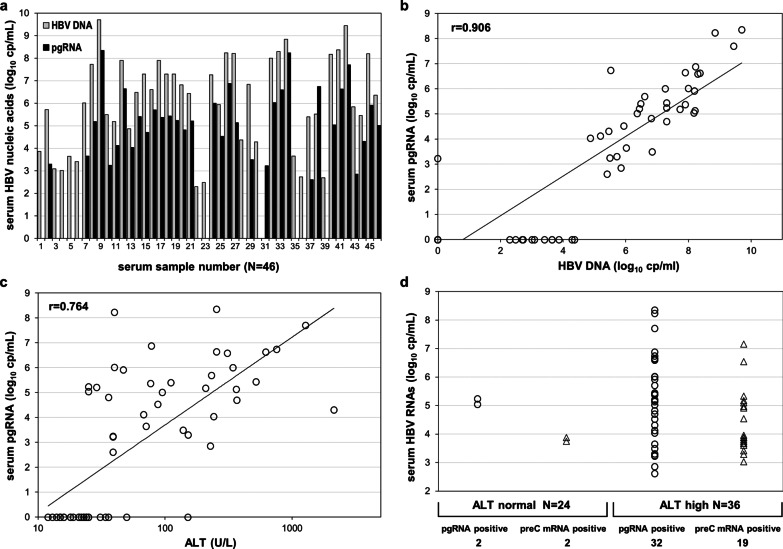
Serum HBV nucleic acid levels in HBeAg negative patients not receiving any treatment. HBV DNA and pgRNA levels in 46 serum DNA and/or RNA positive samples (**a**). Correlation of serum pgRNA with HBV DNA levels (**b**). Correlation of serum pgRNA with ALT levels (**c**). Serum HBV pgRNA (circles) and preC mRNA (triangles) detectability and levels in untreated patients with normal and elevated (as per AASLD) ALT levels (**d**)

HBV DNA was detected in 45/60 (75%) sera, in levels exceeding those of pgRNA by 1.74 (-3.2–3.35) log_10_ cp/mL in 32/34 (94%) of pgRNA-positive samples (Fig. 2a). In the 26 pgRNA negative samples, HBV DNA was either undetectable (n = 14) or measurable (n = 12) at low levels, 3.25 (2.3–4.37) log_10_ cp/mL. Serum pgRNA was detectable in all patients with active CHB (CAHB), with ALT > 2 × upper limit of normal (ULN) and HBV DNA > 5.72 log10 cp/mL, sampled before treatment initiation.

Serum pgRNA strongly correlated to viral replication, expressed by HBV DNA (r = 0.906, *p* < 0.01) and liver damage, expressed by ALT (r = 0.764, *p* < 0.01) (Fig. 2b, c) and was predominantly detectable in samples with ALT above the limits proposed by the American Association for the Study of Liver Diseases (AASLD) (35 IU/L for males and 25 IU/L for females) [16]. From 36 samples with such ALT increase, 32 (89%) had detectable pgRNA compared to only 2 of 24 (8.3%) with normal ALT (Fig. 2d), both HBV DNA positive. Similarly, all samples with detectable preC mRNA had high levels of HBV DNA [7.90 (5.52–9.70) log_10_ cp/mL], whereas 19/21 (90.5%) preC mRNA-positive samples had elevated ALT (Fig. 2d).

### NA treated patients

**Fig. 3:**
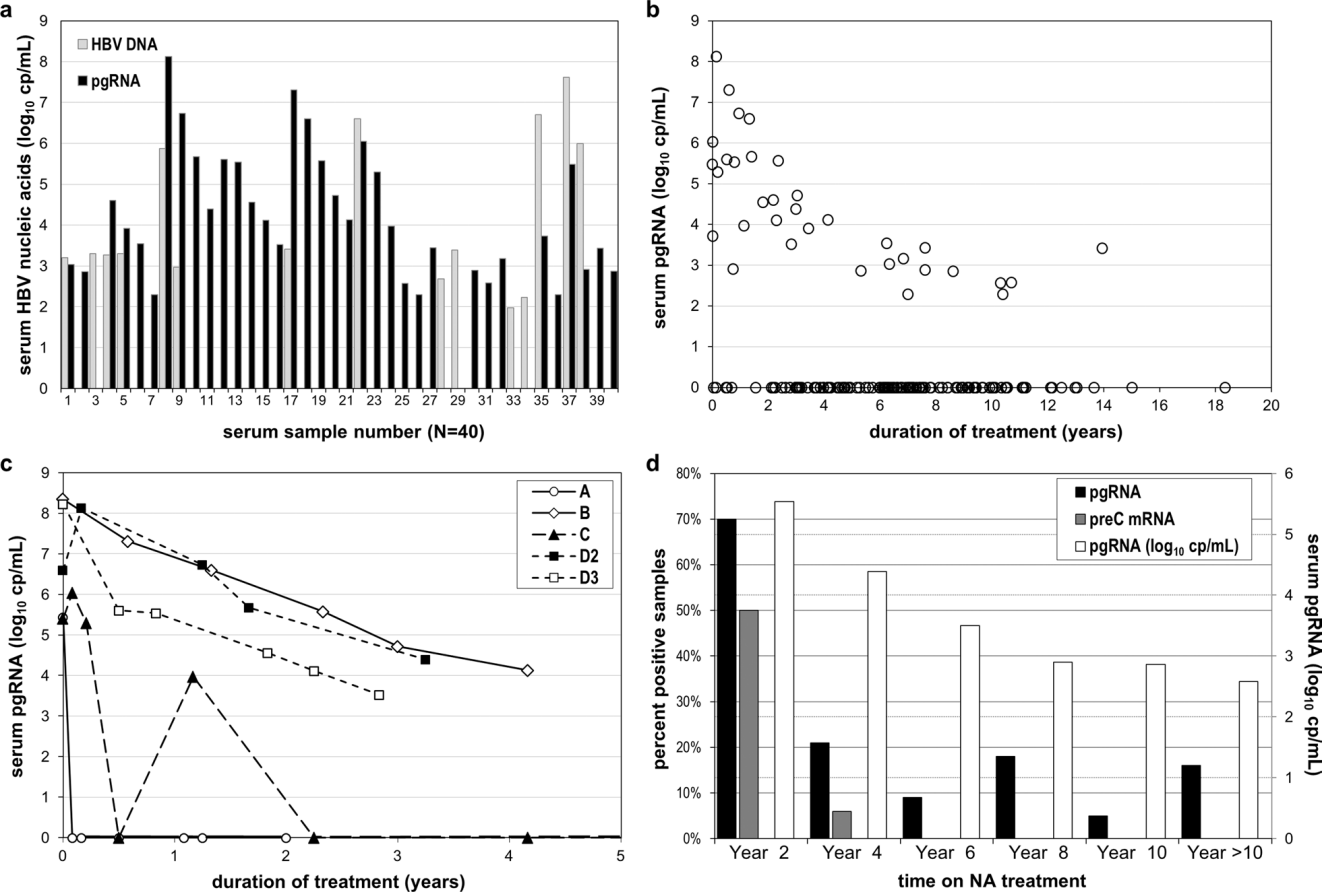
Serum HBV nucleic acid levels in HBeAg negative patients treated with nucleos(t)ide analogues. HBV DNA and pgRNA levels in 40 serum DNA and/or RNA positive samples (**a**). Serum pgRNA levels in relationship to the sampling time (**b**). Longitudinal measurements of serum pgRNA levels in five courses of NA treatment (**c**). Proportion of samples with detectable pgRNA (black), preC mRNA (grey) and corresponding median of detectable pgRNA levels (white) during long term NA treatment (**d**)

HBV pgRNA was found in 35/160 (21.9%) samples and 15/103 (14.5%) patients, at a median level of 3.97 (2.30–8.13) log_10_ cp/mL when detectable, significantly lower than those observed in subjects under no treatment (*p* < 0.01) (Figs. 1 and 3a). Three samples had reproducibly detectable pgRNA below the LQD. PreC mRNA was found in 12 samples, at a median level of 5.64 (3.73–6.78) log_10_ cp/mL, when detectable.

HBV DNA was detectable in 15 (9.4%) samples (11 patients), median level 3.30 (1.97–7.62) log_10_ cp/mL, either in patients close to treatment initiation or during treatment with low genetic barrier NAs. Ten (28.5%) of 35 pgRNA-positive and 5 (4.0%) of 125 pgRNA-negative samples had detectable HBV DNA (Fig. 3a). In the latter 5 samples, HBV DNA ranged from 1.97 to 3.39 log_10_ cp/mL. Altogether, serum HBV RNA showed higher sensitivity than HBV DNA (22% vs 9.4%) in detecting persistence of viral transcriptional activity and was found at higher levels when both markers were detectable, median difference: 3.77 (− 3.08–6.6) log_10_ cp/mL.

We observed a significant inverse correlation of pgRNA levels with the duration of treatment (r = -0.346, *p* < 0.01) (Fig. 3b). Longitudinal pgRNA measurements in five treatment courses are shown in Fig. 3c. A parallel drop on detectability was observed overtime, as pgRNA was detected in 70% (14/20) of samples collected within the first two years compared to 13.2% (21/140) of samples collected after two years of treatment (Fig. 3d). Serum pgRNA was associated with persisting viral activity and liver damage soon after treatment initiation. Out of 10 pgRNA-positive samples collected within the first year of NA treatment, 7 had detectable HBV DNA, 7 increased ALT and 6 had both. PreC mRNA was detected in 8 of these samples.

**Fig. 4:**
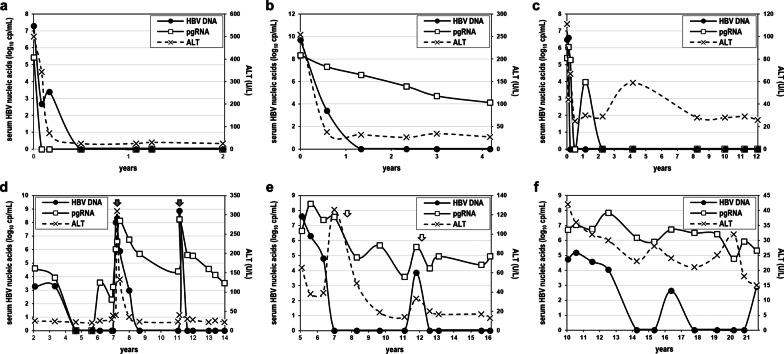
Analysis of archival longitudinal samples of patients under nucleos(t)ide analogue treatment. Longitudinal serum samples of 4 HBeAg negative (**a**–**d**) and 2 HBeAg positive patients (**e** and **f**). NA treatment initiation at time point zero or indicated with closed arrows, pegylated interferon-alfa treatment initiation indicated with open arrows

To investigate the long-term kinetics of serum pgRNA during NA treatment, we analyzed archival longitudinal samples from 5 patients, spanning 4–15 years of observation. Two patients, who were pgRNA negative during treatment, achieved HBsAg loss during post-treatment follow-up, one case is depicted in Fig. 4a. One patient, still under treatment, was persistently pgRNA positive during four years of observation (Fig. 4b), while another was initially pgRNA positive until ALT normalization and subsequently negative (Fig. 4c). A more complex profile was observed in the fifth patient during a 12-year observation period, encompassing two failed attempts of NA discontinuation. During the first course of treatment, pgRNA transiently fell twice below the LLD and was reproducibly detectable below the LQD on one occasion, while all tested samples were pgRNA positive throughout the second and third treatment course (Fig. 4d).

For comparison, we examined 24 samples from two, rare in Greece, genotype A HBeAg positive patients, 12 longitudinal samples from each spanning 11 years of NA treatment (Fig. 4e, f). HBV RNA was consistently detectable, with median pgRNA levels of 6.07 (3.58–8.44) log_10_cp/mL being significantly higher than those of HBeAg negative NA treated patients (*p* < 0.001). In one patient addition of pegylated interferon-alpha (Peg-IFNa) to NAs twice resulted in significant reduction of pgRNA levels (2.7 and 1.4 log_10_ cp/mL). PreC mRNA was detected in 15 (63%) samples, at a median of 5.06 (3.42–6.70) log_10_ cp/mL and similarly to HBeAg negative patients, constituted a small fraction (2.5%) of CP-produced transcripts.

### NA discontinuation

The observed diversity in the serum pgRNA profile among HBeAg negative patients under NAs, taken together with the large number of pgRNA negative samples, pose a question as to whether serum pgRNA can be a useful predictive marker for NA discontinuation. We analyzed 74 end of treatment (EOT) samples from 73 patients with long-term post-treatment follow-up (one patient had two attempts of NA discontinuation). All EOT samples were HBV DNA negative, with normal ALT levels. VR was defined as HBV DNA > 2000 IU/mL (10,000 cp/mL) within one year after NA discontinuation. CR was defined as combination of VR with ALT > 2xULN.

The majority of samples 67/74 (90.5%) had undetectable pgRNA, which was detected only in 7 (9.5%) samples at a median level of 2.58 (2.3–4.39) log_10_ cp/mL, including samples with reproducibly detectable pgRNA below the LQD. NA discontinuation led to VR in 35/67 (52.2%) EOT pgRNA negative and in all 7 (100%) pgRNA positive cases (*p* = 0.016) and to CR in 17/67 (25.4%) pgRNA negative compared to 5/7 (71%) pgRNA positive cases (*p* = 0.011). Two patients with low EOT pgRNA (750 and < 350 cp/mL) experienced only VR and remained in clinical remission for 46 and 37 months of follow-up. For the remaining 5 patients with detectable pgRNA, VR was rapidly followed (0–2 months) by CR. Of the 17 EOT pgRNA negative patients with CR, 9 (53%) were retreated within the first year, while all 5 (100%) EOT pgRNA positive patients with CR required retreatment (*p* = 0.001, HR = 6.569, 95% CI 2.194–19.669).

HBsAg clearance was observed in 33 (49.3%) pgRNA negative patients at a median of 12 (range 0–96) months of post-treatment follow-up, compared to none of the 7 pgRNA positive patients (*p* = 0.013) (Table 2). Anti-HBs was detected in 21 (64%) of the 33 patients with HBsAg clearance at median time of 15 months (range 0–26) after HBsAg loss and median titer 102 mIU/mL (range 12–943). All pgRNA positive samples had qHBsAg > 100 IU/mL, but no significant difference between pgRNA positive and pgRNA negative samples in qHBsAg levels (*p* = 0.297), ALT (*p* = 0.432) or duration of treatment (*p* = 0.183) was found. (Table 2).

**Table 2:** Patient characteristics at NA discontinuation and outcomes in relation to the detectability of pgRNA at EOT

	Total N = 74	pgRNA positive N = 7	pgRNA negative N = 67	*p* value
*Patient characteristics at NA discontinuation*				
Age, years	60 (27–83)	64 (43–73)	60 (27–83)	0.561
Sex, females/males	19/55	2/5	17/50	0.855
NA genetic barrier, high/low	14/60	7/0	53/14	0.179
Duration of NA treatment, months	75 (36–220)	94 (36–167)	74 (36–220)	0.183
ALT, IU/L	21 (10–40)	23 (12–40)	21 (10–40)	0.432
HBsAg, log_10_ IU/mL	2.48 (ND–4.59)	2.73 (2.03–3.77)	2.3 (ND–4.59)	0.297
*Outcomes after NA discontinuation*				
Virological relapse, n (%)	42 (57)	7 (100%)	35 (52%)	**0.016**
Clinical relapse, n (%)	21 (28)	5 (71%)	17 (25%)	**0.011**
Retreatment, n (%)	14 (19)	5 (71%)	9 (13%)	** < 0.01**
HBsAg loss, n (%)	33 (45)	0	33 (49%)	**0.013**

Quantitative variables are presented as median (min–max) values, in bold statistically significant values (*p* < 0.05) are shown

NA, nucleos(t)ide analogue; ND, not detectable

Post-treatment VR was independently associated with higher EOT pgRNA levels (HR per log_10_: 1.524, 95% CI: 1.132–2.051; *p* = 0.006,) and qHBsAg (HR per log_10_IU/mL: 1.391, 95% CI: 1.023–1.891; *p* = 0.035), while CR was significantly associated with higher EOT pgRNA (HR per log_10_: 1.569, 95% CI: 1.126–2.187; *p* = 0.008) and older age (HR per year: 1.048, 95% CI: 1.002–1.095; *p* = 0.039). HBsAg clearance was associated only with lower EOT qHBsAg (HR per log_10_IU/mL: 0.241, 95% CI: 0.161–0.360; *p* < 0.001).

### Stoichiometry and correlations

**Fig. 5:**
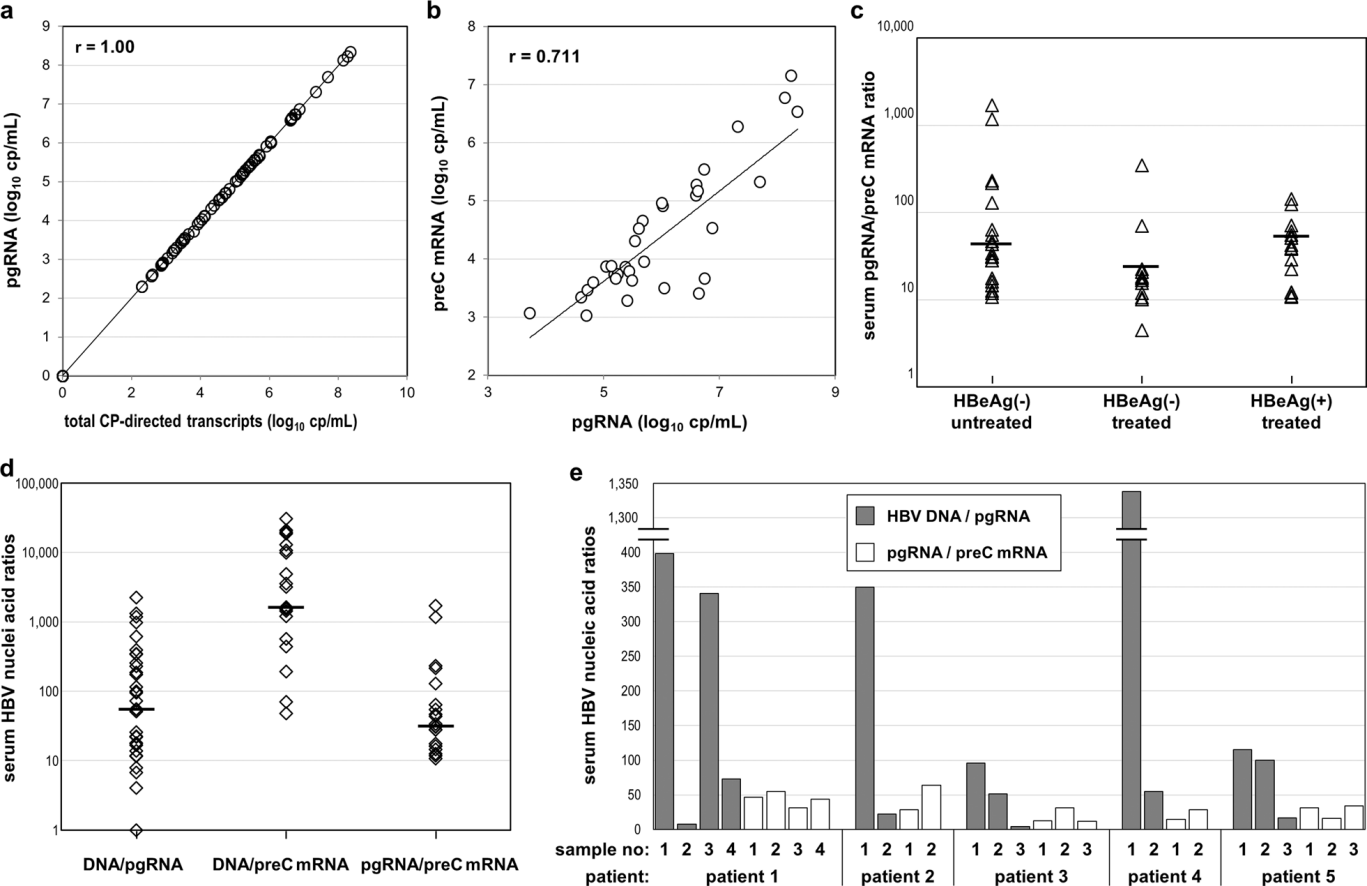
Serum HBV nucleic acid correlations and ratios. Serum HBV RNA species correlations in all HBeAg negative patients (**a** and **b**) and pgRNA to preC mRNA ratio in all patients (**c**). Serum HBV nucleic acid ratios in untreated patients, all samples tested (**d**) and consecutive serum samples from five patients (**e**)

As preC mRNA represented a small fraction of total HBV RNA and CP-directed transcripts mostly reflect pgRNA levels, there was an extremely high correlation between total HBV RNA and pgRNA (r = 1.000, *p* < 0.01) (Fig. 5a). In considering the relationship between HBV RNA species, preC mRNA showed strong correlation with pgRNA (r = 0.711, *p* < 0.01) in all HBeAg negative samples (Fig. 5b), though weaker during NA treatment (r = 0.622, *p* < 0.01). For the majority of preC mRNA positive samples (39/48), the relative amount of CP-directed transcripts (pgRNA/preC mRNA) ranged between 10 and 100, with median values of 31.5 for patients not receiving treatment, 17 for NA treated HBeAg negative patients and 39 for NA treated HBeAg positive patients (Fig. 5c).

For patients not receiving treatment, it is meaningful to evaluate the relative amount of serum HBV DNA to that of HBV RNA species. A wider variation of the relative amount of HBV DNA to either RNA species was observed (0.1–2,200 for DNA/pgRNA and 50–30,000 for DNA/preC mRNA), than when compared to each other, pgRNA to preC mRNA (10–1700) (Fig. 5d). Furthermore, in a limited number of consecutive samples of patients not receiving treatment, for each patient, the pgRNA/preC mRNA ratio was found to remain relatively constant, maximum variation of 1.7- to 2.6-fold, compared to significant changes, 9- to 50-fold, of the HBV DNA/pgRNA ratio (Fig. 5e).

**Fig. 6:**
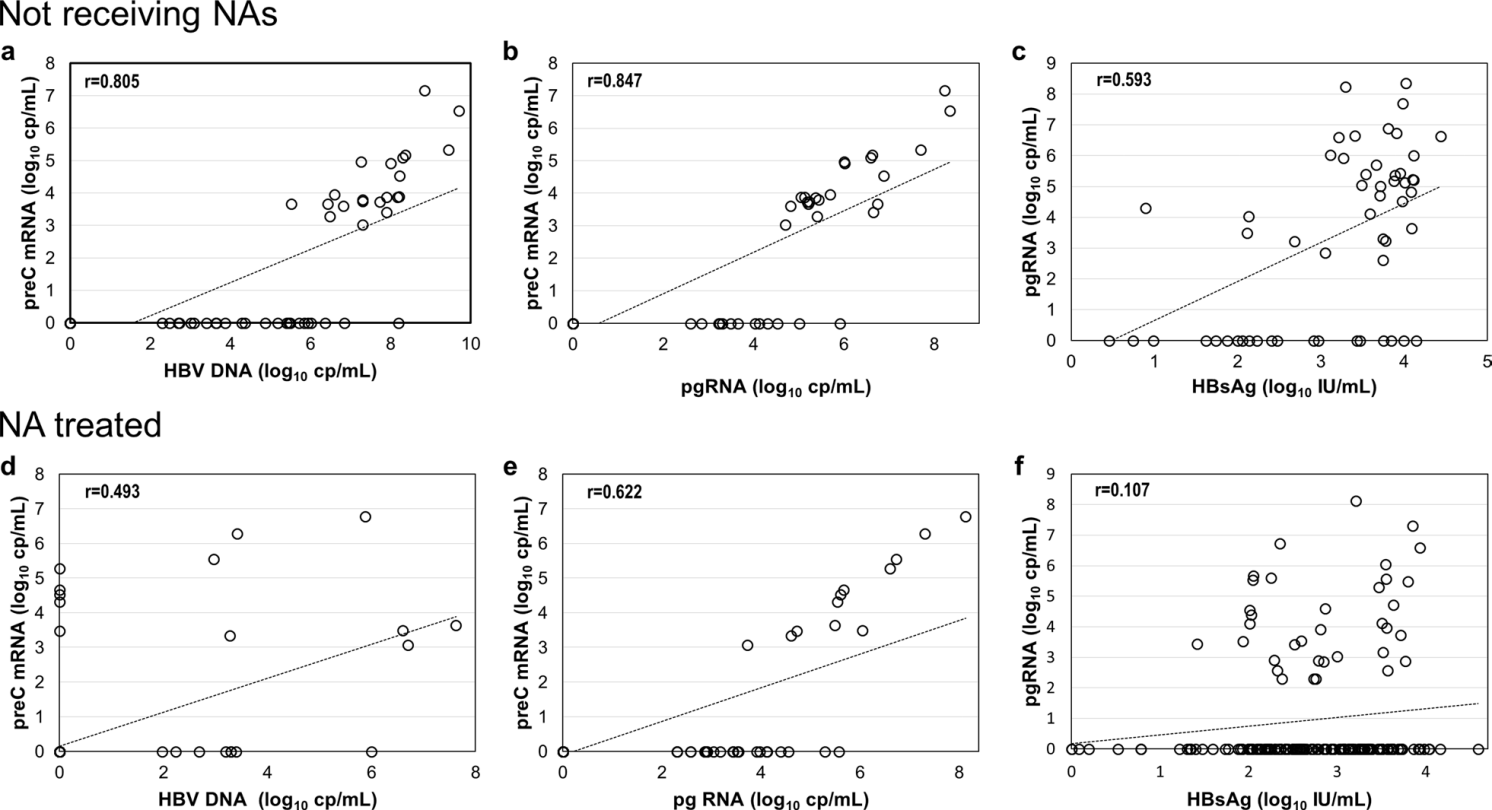
Correlations of HBV nucleic acids and HBsAg. Correlations of levels of serum HBV nucleic acids and HBsAg in HBeAg negative patients not receiving any treatment (**a**–**c**) and patients treated with nucleos(t)ide analogues (**d**–**f**)

The levels of either pgRNA or preC mRNA correlated well to HBV DNA levels in patients not receiving NAs (r = 0.906 and r = 0.805, respectively, *p* < 0.01) (Figs. 2b and 6a), while both species showed weaker correlation with HBV DNA levels during NA treatment (r = 0.391 and r = 0.493, *p* < 0.01). In subjects not receiving NAs, pgRNA correlated with ALT (r = 0.764, *p* < 0.01) and weaker with qHBsAg (r = 0.593, *p* < 0.01) (Fig. 6c). In NA treated patients, no significant correlation was found with qHBsAg levels (r = 0.107, *p* = 0.178) (Fig. 6f) and the correlation with ALT was less tight (r = 0.31, *p* < 0.01)) than that of subjects not receiving treatment.

## Discussion

Intrahepatic production of pgRNA reflects the transcriptional and replicative activity of cccDNA, however secretion of HBV RNA containing particles in the serum is not a canonical pathway in the HBV life cycle and the mechanism and conditions that lead to their production and egress are largely unknown. The precise nature of serum HBV RNA, the presence of free HBV RNA molecules, the composition and origin of detected HBV RNA, encapsidated or not, are some of the important issues that remain unresolved.

Serum HBV RNA has been proposed as a viral biomarker of intrahepatic cccDNA activity, particularly for treatment response and safe NA discontinuation [13, 17–21]. However, studies concerning genotype D ab initio HBeAg negative patients are limited and more difficult to interpret. The globally widespread HBV genotype D overwhelmingly (> 99%) prevails among native Greeks [22], in most (> 98%) cases harbouring the G1896A precore mutation, plus a wide range of additional mutations that accumulate in the basic core promoter region during the course of infection [23, 24]. These mutations affect HBV gene expression, replication, and perhaps pathogenicity by changing the intrahepatic levels and balance between pgRNA and preC mRNA production and the transcriptional activity of HBV cccDNA molecules [14, 15, 25–27]. Such alterations may have an impact on the levels and detectability of HBV transcripts in the serum and thus affect the clinical value of serum HBV RNA as a biomarker in this population.

Our study, focusing on this major, yet discrete group of patients, provides novel information concerning three important issues: (1) The composition and origin of serum HBV transcripts, (2) The detectability and levels of serum pgRNA in untreated and NA treated patients and (3) The value of serum HBV RNA as a predictive indicator of relapse after NA discontinuation.

Our methodology for the quantification of serum HBV RNA records both pgRNA and preC mRNA levels. Serum HBV RNA was found to be primarily composed of pgRNA, as previously described [9]. However, we found preC mRNA in all samples in which total HBV RNA levels were sufficient to allow its detection, given its low representation and the assay’s LLD. Until now, there have been conflicting reports regarding the presence of preC mRNA in serum [8, 9, 28]. Our data unequivocally demonstrate that preC mRNA is a minor but integral component of serum HBV RNA molecules.

Since preC mRNA is not incorporated into nucleocapsids, its detection in serum may represent free molecules originating from damaged hepatocytes or erroneous encapsidation and either illegitimate export, as argued for pgRNA, or release from lysed cells. We found the presence of preC mRNA to be associated with both serum HBV DNA and elevated ALT levels. The ratio of pgRNA to preC mRNA in 30 of 33 samples ranged between 10 and 3 × 10^2^, which is similar to the intrahepatic ratio previously reported by our group [15]. This could imply either that there is no stringent selective packaging for pgRNA or that serum ribonucleic acids are, at least in part, of intracellular origin. However, different mutation combinations found in the HBV CP-precore region in HBeAg negative patients, may or may not affect the pgRNA/preC mRNA balance. Therefore, it is essential to know the CP-precore sequences and more important, both serum and synchronous liver biopsy samples must be analyzed. In such a study, investigators reported a 6.6-fold lower representation of preC mRNA in serum than in the liver, but this may reflect differences in encapsidation status and stability of the two species [28]. The inclusion of serum HBV transcripts in nucleocapsids does not demonstrate their origin to be solely nucleocapsid export, as immune lysis of infected cells would release both encapsidated and free transcripts into the blood stream, the latter being more sensitive to degradation both in vivo and in vitro.

Our data from subjects not receiving treatment showed a wide range of HBV DNA/pgRNA ratio (< 1 to > 1000) among different samples and significant variation among samples from the same patient ranging 7- to 50-fold. These results are inconsistent with the proposed integral mechanism of exporting pgRNA containing particles along with mature rcDNA containing virions as a mechanistically linked innate aspect of HBV replication [29], and suggest that either illegitimate export of encapsidated pgRNA is an erratic process or other factors, such as immune damage of infected cells, contribute to the serum HBV nucleic acid pool. Indeed, our findings indicate lysis of infected hepatocytes as an alternative explanation for the origin of serum HBV ribonucleic acids by showing strong correlation with disease activity and liver damage. In patients not receiving treatment, pgRNA strongly associated with viral replication and was also predominantly (94%) detected in samples with high ALT. Previous reports also recorded a significant correlation of HBV RNA with ALT and/or viral replication [30–32], yet immune mediated lysis of hepatocytes was not considered as a significant source of serum HBV RNA. On the contrary, liver cell damage has been generally accepted to be the source of serum cccDNA [33, 34].

Additionally, our study suggests that serum pgRNA levels may have a dual origin in the early phase of treatment. In the first year of NA treatment, serum pgRNA was associated with persisting viral replication and liver damage. Thus, it appears that immune lysis of infected cells contributes to serum HBV nucleic acid content early in NA treatment, a process that is quickly restricted and may, in part, explain the steep decline of the first phase of serum HBV RNA biphasic kinetics [35].

Our work has a few limitations regarding the origin of serum HBV RNA transcripts that must be addressed. Liver biopsy tissue was not available in order to have an intrahepatic frame of reference for pgRNA/preC mRNA ratio for each sample and our methodology does not discriminate between encapsidated and naked HBV RNA. The evidence for immune lysis of hepatocytes as a source of serum HBV RNA is based on clinical data and requires direct confirmation with other experimental approaches. Our preliminary data (not shown) demonstrate that RNase treatment of sera prior to RNA extraction results in reduced amounts of both pgRNA and preC mRNA in samples with high ALT levels.

We evaluated the detectability and levels of serum pgRNA in samples from untreated and patients under NA treatment. HBV pgRNA was detected in 57% of samples collected under no treatment, which is intermediate to rates of 77% and 22% reported in two recent studies [30, 36]. These differences can be attributed to a variety of host and viral factors, such as genotype, CP mutations and the sensitivity and specificity of the assays [30, 37]. In the absence of NAs, HBV DNA was detectable more frequently and at higher levels compared to pgRNA, thus serum HBV RNA provides no additional information on viral activity. Although serum pgRNA correlated strongly to ALT and HBV DNA levels, all untreated CHB patients were pgRNA positive, while pgRNA was detectable in only one IC with detectable HBV DNA. These findings are also in agreement with the association between serum HBV RNA and the phases of untreated chronic HBV infection [17, 38].

In NA treated patients, serum HBV RNA has been associated with cccDNA intrahepatic transcriptional activity and liver histology [39, 40]. A major obstacle to its clinical application in HBeAg negative CHB is its low detectability rate, indeed, we detected pgRNA in only 22% of samples and 20% of patients undergoing NA treatment. Moreover, the proportion of positive samples and the corresponding pgRNA levels depended on the duration of treatment, dropping from 70 to 15% after the second year and declining by more than 2 log_10_ cp/mL. Similarly, only 14% of patients were recently reported to remain serum HBV RNA positive after 5 years of effective NA treatment [41].

In our group of NA treated patients, serum pgRNA did not follow a consistent or predictable pattern, but displayed significant variability, even considering the same patient. It was found to be periodically or persistently detectable at high or low levels, to fluctuate above and below the LLQ or more frequently, not to be detectable at all. This can be partly attributed to the fact that in these patients, long-term NA treatment results in intrahepatic cccDNA and pgRNA levels that border the limits of detectability [10, 11]. The low detectability in our HBeAg negative CHB patients under NA treatment is in striking contrast to our findings in HBeAg positive patients who, in agreement with existing reports, presented high pgRNA levels in all samples [29, 30, 38, 40, 42, 43]. Even so, serum pgRNA can be a valuable indicator of residual cccDNA activity during NA treatment of HBeAg negative CHB, as it was detected in twice as many samples and at higher levels compared to serum HBV DNA.

Clinical application of serum HBV RNA, as a biomarker for monitoring response to treatment and predicting safe NA cessation, is the most important aspect under evaluation. Our study included a large number of EOT samples, in which pgRNA positivity was less than 10% and when detected, pgRNA levels were low, often bordering the LLD. Given that pgRNA could be detectable or not, even in the same patient, during the course of NA treatment, analysis of a single sample may not suffice.

We found detectability of pgRNA in patients under long-term effective NA to have high clinical relevance, as it predicted VR after NA discontinuation in all our cases. Moreover, there was significant association of EOT pgRNA detectability with 1-year probability of CR and retreatment. Conversely, 25% of patients with undetectable pgRNA experienced CR, suggesting the necessity of more sensitive assays, repetitive tests and/or combining pgRNA with other viral markers, such as qHBsAg and/or HBcrAg, in order to increase the accuracy of predictability. In fact, recent reports demonstrated that combination of viral markers is highly predictive of CR after NA discontinuation [41, 44, 45].

Among EOT pgRNA negative patients, 49% achieved HBsAg clearance during post-NAs follow-up, predicted by low EOT qHBsAg, as previously reported [46, 47]. From 12 patients with EOT qHBsAg < 10 IU/mL all HBV RNA negative, only one experienced transient VR and all ultimately cleared HBsAg, as observed in a recent study [48]. Thus, combining qHBsAg and serum HBV RNA could be a valuable approach for NA treatment monitoring and strategies of discontinuation.

## Conclusions

Our study on genotype D ab initio HBeAg negative patients, demonstrates that serum HBV RNA is primarily composed of pgRNA plus a minor but integral fraction of preC mRNA. The association of serum pgRNA with disease activity, suggests lysis of infected hepatocytes as a possible source of HBV transcripts which may contribute to the serum HBV RNA pool in subjects not receiving treatment and in the early stage of NA therapy. During the natural course of chronic HBV infection, serum pgRNA is a consistent marker of viral replication and disease activity. Despite limited detectability and low levels, serum pgRNA could be a helpful marker in NA discontinuation strategies, as its presence at EOT is a definite predictor of VR and is strongly associated with subsequent CR and retreatment.

## Data Availability

The data analyzed in the current study is available from the corresponding author on reasonable request.

## Abbreviations

HBV: Hepatitis B virus
cccDNA: Covalently closed circular DNA
HBeAg: Hepatitis B e antigen
pgRNA: Pregenomic RNA
preC mRNA: Precore mRNA
NA: Nucleos(t)ide analogue
CHB: Chronic hepatitis B
HCC: Hepatocellular carcinoma
Peg-IFNa: Pegylated interferon-alfa (Peg-IFNa)
HBV CP: HBV core promoter
rcDNA: Relaxed circular DNA
RT-PCR: Real time polymerase chain reaction
IC: Inactive carriers
EOT: End of treatment
HBsAg: Hepatitis B surface antigen
cp: Copies
LLD: Lower limit of detection
LQD: Limit of quantitative detection
ALT: Alanine aminotrasferase
VR: Virological relapse
CR: Clinical relapse
IU: International units
ULN: Upper limit of normal
